# 4D-DIA Proteomics Uncovers New Insights into Host Salivary Response Following SARS-CoV-2 Omicron Infection

**DOI:** 10.1021/acs.jproteome.4c00630

**Published:** 2025-01-13

**Authors:** Iasmim Lopes de Lima, Thais Regiani Cataldi, Carlos Brites, Mônica Teresa Veneziano Labate, Sara Nunes Vaz, Felice Deminco, Gustavo Santana da Cunha, Carlos Alberto Labate, Marcos Nogueira Eberlin

**Affiliations:** † PPGEMN, School of Engineering, Mackenzie Presbyterian University & MackGraphe - Mackenzie Institute for Research in Graphene and Nanotechnologies, Mackenzie Presbyterian Institute, São Paulo, São Paulo 01302-907, Brazil; ‡ Department of Genetics, “Luiz de Queiroz” College of Agriculture, University of São Paulo/ESALQ, Piracicaba, São Paulo 13418-900, Brazil; § LAPI - Laboratory of Research in Infectology, University Hospital Professor Edgard Santos (HUPES), Federal University of Bahia (UFBA), Salvador, Bahia 40110-060, Brazil

**Keywords:** Omicron, saliva, proteomics, mass spectrometry, COVID-19, SARS-CoV-2, DIA-PASEF

## Abstract

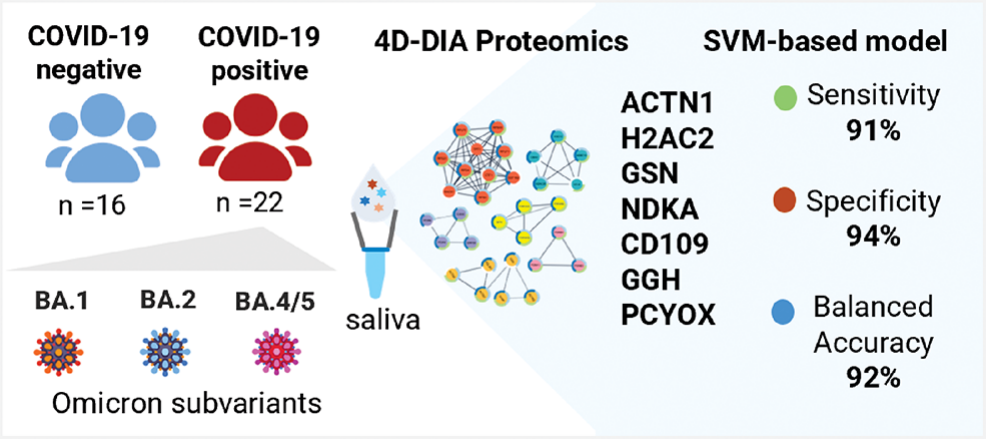

Since late 2021, Omicron variants have dominated the epidemiological scenario as the most successful severe acute respiratory syndrome coronavirus 2 (SARS-CoV-2) sublineages, driving new and breakthrough infections globally over the past two years. In this study, we investigated for the first time the host salivary response of COVID-19 patients infected with Omicron variants (BA.1, BA.2, and BA.4/5) by using an untargeted four-dimensional data-independent acquisition (4D-DIA)-based proteomics approach. We identified 137 proteins whose abundance levels differed between the COVID-19 positive and negative groups. Salivary signatures were mainly enriched in ribosomal proteins, linked to mRNAviral translation, protein synthesis and processing, immune innate, and antiapoptotic signaling. The higher abundance of 14-3-3 proteins (YWHAG, YWHAQ, YWHAE, and SFN) in saliva, first reported here, may be associated with increased infectivity and improved viral replicative fitness. We also identified seven proteins (ACTN1, H2AC2, GSN, NDKA, CD109, GGH, and PCYOX) that yielded comprehension into Omicron infection and performed outstandingly in screening patients with COVID-19 in a hospital setting. This panel also presented an enhanced anti-COVID-19 and anti-inflammatory signature, providing insights into disease severity, supported by comparisons with other proteome data sets. The salivary signature provided valuable insights into the host’s response to SARS-CoV-2 Omicron infection, shedding light on the pathophysiology of COVID-19, particularly in cases associated with mild disease. It also underscores the potential clinical applications of saliva for disease screening in hospital settings. Data are available via ProteomeXchange with the identifier PXD054133.

## Introduction

The SARS-CoV-2 Omicron variant (B.1.1.529) and its sublineages became globally dominant by the end of 2021, causing a series of new and breakthrough infections characterized by asymptomatic and mild-to-moderate symptoms..^1^ Although associated with low pathogenicity, the Omicron variant is highly transmissible, leading to multiple infection waves in several countries across recent years.^2,3^ Compared to previous lineages, Omicron subvariants are more likely to evade immunity acquired from prior SARS-CoV-2 infections and vaccinations,^4,5^ increasing reinfection cases.

Despite its relatively low pathogenicity—ascribed to reduced lung tropism^6^—the variant’s transmission and escape from antibody neutralization are mainly facilitated by numerous mutations in the receptor-binding domain (RBD) of the spike protein, which binds to the host’s angiotensin-converting enzyme 2 receptor (ACE2).^7^ Recent investigations indicate that RBD sites are under strong diversifying selection, indicating ongoing viral diversification via mutations.^8^ Indeed, despite increased hybrid immunization, new descendants of Omicron variants have been associated with escape from vaccine-mediated immune protection, higher viral fitness, and additional immune escape properties.^8−11^

Omicron surge has generally reduced hospitalizations, yet it has increased pediatric hospital admissions,^12^ and hospital-onset SARS-CoV-2 infections, including nosocomial transmission,^13,14^ posing therefore risks to vulnerable populations, such as older adults and immunosuppressed individuals.^15^ Patients infected with SARS-CoV-2 Omicron variants also had a higher risk of severe disease and in-hospital mortality than those infected with influenza and interstitial viruses.^16,17^ Therefore, understanding the host response to a new infection profile can aid in understanding its pathophysiological mechanisms and guide appropriate clinical management, including therapeutic strategies.

A previous study highlighted that different SARS-CoV-2 variants can induce distinct metabolic dysregulation in the host.^18^ Li et al. also showed that Omicron infections mainly promote innate immunity while suppressing adaptive immunity, activating unique metabolic pathways unseen with the original SARS-CoV-2 strain.^19^ A blood-based multiomics analysis further revealed an enhanced interferon-mediated antiviral signature in platelets from Omicron-infected patients, as well as activation of the coagulation cascade and hemostatic mechanisms.^20^ Additionally, Omicron breakthrough infections have been linked to a reduced number of B cells and a limited systemic inflammatory response.^20^

Besides systemic alterations in blood, changes in salivary molecules also occur in response to SARS-CoV-2 infection.^21−25^ Salivary glands have been identified as a reservoir and replication site for the virus,^26^ making saliva a key transmission vector,^27^ which affects both salivary content and production, inducing important oral manifestations.^28,29^ Saliva collection is also cost-effective and noninvasive, making it a viable alternative to more invasive sampling methods for molecular diagnosis of infectious diseases.^30,31^ This approach also serves as a promising alternative to blood in monitoring COVID-19 inflammatory responses,^32^ offering a convenient method to assess disease status while providing insights into COVID-19 severity and progression.^32,33^

Salivary proteomics has provided several insights into SARS-CoV-2 infection, linking remarkable protein changes to altered taste perception, respiratory complications, and increased susceptibility to viral infection.^21^ Saliva has also proven effective in identifying and monitoring abnormal and exacerbated COVID-19 immune responses, including vascular, inflammatory, and coagulation-related sequelae.^22,34^

Mass spectrometry-based proteomics is, therefore, a valuable approach for identifying molecular signatures related to the pathophysiology of COVID-19. This technique not only aids in understanding the disease but also helps in screening molecules for potential use in point-of-care devices and analytical platforms, enabling multiplexing and facilitating rapid and large-scale disease screening and monitoring.^35^ However, proteomics studies involving saliva remain limited compared to other matrices such as serum and plasma, and the host-specific salivary signature following Omicron infection has not been fully established.

In this study, we have focused on the salivary host response of hospital-recruited patients with COVID-19 infected with SARS-CoV-2 Omicron BA.1, BA.2, or BA.4/5 variants versus controls with COVID-19-like symptoms. To investigate the salivary protein profile of early to-acute SARS-CoV-2 infection, we employed a four-dimensional data-independent acquisition-parallel accumulation-serial fragmentation (4D DIA-PASEF) proteomic approach. Driven by the promissory clinical applications of saliva,^36^ we also investigate if salivary proteins could differentiate between COVID-19-positive and negative individuals with similar phenotypes in a hospital setting. Although RT-PCR remains the gold standard for COVID-19 diagnosis, the development of new screening approaches is especially relevant in an endemic context with a constantly evolving virus. Some antigen tests have also shown a lower performance, especially after the circulation of the Omicron subvariants, and antibody tests are not recommended for disease screening due to the higher overall seroprevalence in the population.^37−39^

Our findings highlight the activation of multiple mechanisms related to the pathophysiology and clinical manifestations of the disease, providing new insights into Omicron infections. We identified blood-based markers of COVID-19^40−42^ in saliva samples, suggesting that the salivary signature may reflect the systemic host response and emphasizing its potential clinical applications.

## Experimental Section

### Study Population and Experimental Design

From January to July 2022, passive self-collected saliva samples were obtained from 38 volunteers, including outpatients and healthcare professionals, who were suspected of having or had been exposed to COVID-19 at the Complexo Hospital Professor Edgard Santos (C-HUPES) of the Federal University of Bahia in Salvador, Brazil. The study was conducted following principles outlined in the Declaration of Helsinki and was approved by the Research Ethics Committee of the Climério de Oliveira Maternity Unit at the Federal University of Bahia (UFBA) (protocol number 31748320.3.0000.5543, dated 05/22/2020). Demographic and clinical information were collected, along with saliva samples, after all volunteers signed an informed consent form.

**Figure 1:**
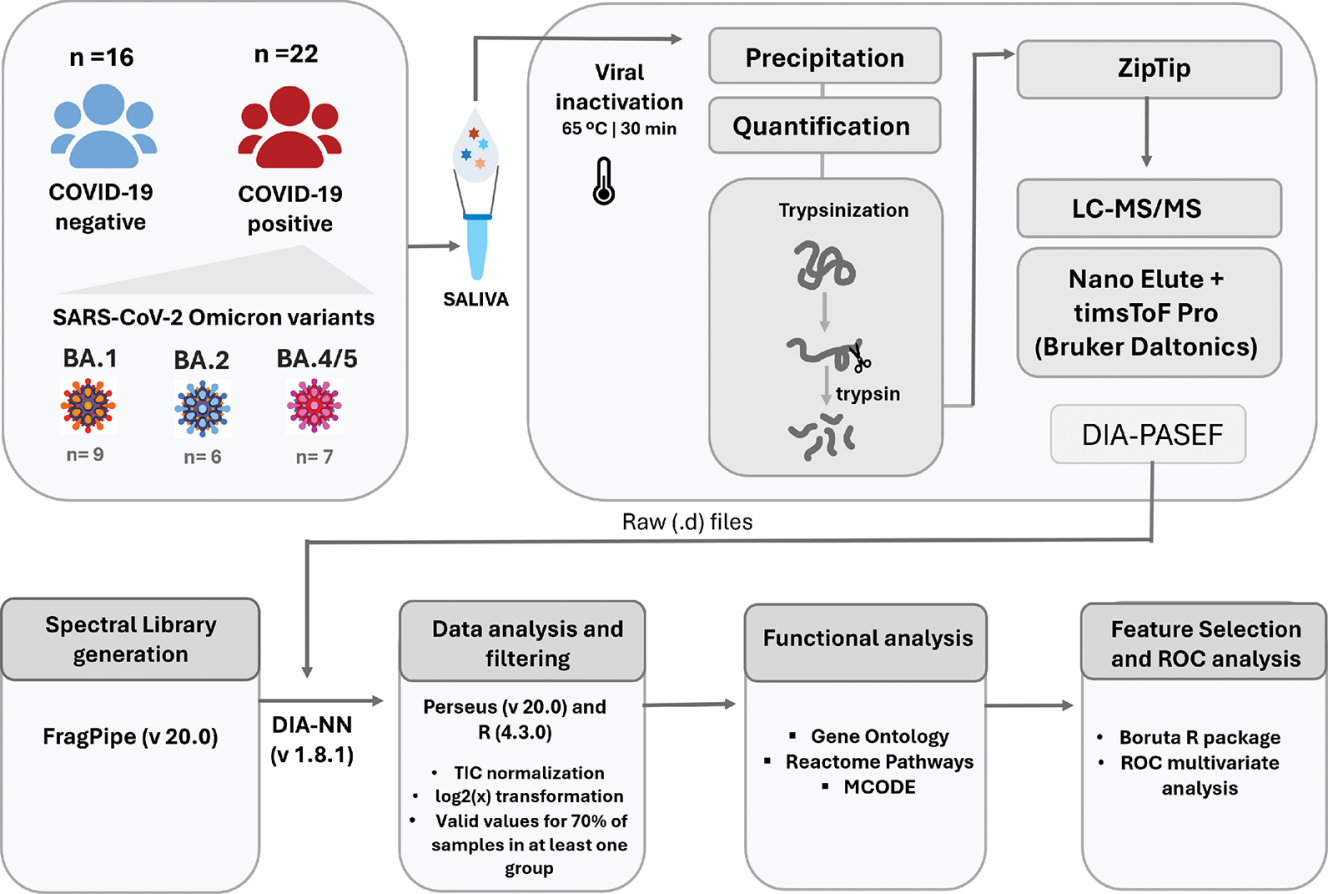
Schematic representation of the study design and proteomics workflow. LC-MS/MS: Liquid Chromatography-Tandem Mass Spectrometry; DIA-PASEF: data-independent acquisition-parallel accumulation-serial fragmentation; TIC: total ion chromatogram. ROC: receiver operating characteristic.

All participants had their diagnosis confirmed by SARS-CoV-2 RT-qPCR following the Charité-Berlin protocol recommended by WHO,^43^ and validated for saliva samples by Vaz et al.^31^ The maximum interval between symptom onset and saliva collection was 14 days. To classify disease severity, we used COVID-19 guidelines from the National Institutes of Health (NIH, MD, USA).^44^ The RT-qPCR-positive samples were sequenced following the protocol described by Deminco et al. to identify SARS-CoV-2 variants.^45^Table S1 lists the volunteer’s metadata, whereas Figure 1 shows a schematic representation of the study design and the proteomics workflow.

### Saliva Collection

Self-collection of saliva was performed as per the protocol described by Vaz et al.^31^ Food restrictions and the use of cream or mouthwash were recommended for at least 30 min prior to collection. The participants were instructed to spit approximately 2 mL of saliva into sterile tubes. Samples were homogenized, diluted with 1× phosphate-buffered saline (PBS, 1:1, v/v) for RT-qPCR test, and stored at −80 °C. Prior to proteomics analysis, saliva aliquots were thermally inactivated (65 °C, 30 min) and centrifuged (10,000 × g at 4 °C for 10 min). All steps were performed according to the biosafety guidelines and regulations.

### Protein Precipitation and Tryptic Digestion

Protein precipitation was performed according to Hurkman and Tanaka (1986)^46^ with some minor modifications. Briefly, 1.3 mL of 0.1 M ammonium acetate in methanol (100%) was added to 100 μL of saliva and incubated overnight at −20 °C. The samples were then centrifuged at 16,000 × g for 30 min at 4 °C, and the resulting pellets were washed twice with 0.1 M ammonium acetate in methanol (100%) and once with ice-cold acetone (100%). Samples were kept at −20 °C for 1 h and centrifuged at 16 000 × *g* for 30 min at 4 °C. Dried precipitates were then resuspended in 400 μL of rehydration buffer containing 7 M urea, 2 M thiourea, 10 mM dithiothreitol (DTT, cat. no. 161–0611, Bio-Rad), and 0.01% Triton X-100. Protein extracts were desalted using Amicon Ultra-0.5 mL 3 kDa NMWL filters (cat. no. UFC5003BK, Millipore, Billerica, MA, USA) and then reconstituted in a 50 mM NH_4_HCO_3_ solution. Total proteins were quantified using the Bradford method.^47^

Approximately 10 μg of protein from each sample was denatured in 0.2% RapiGest SF (cat. no. 186001861, Waters Corporation, Milford, MA, US) at 80 °C for 15 min, reduced with 2.5 μL of 100 mM DTT at 60 °C for 30 min, and alkylated with 2.5 μL of 300 mM iodoacetamide (IAA, cat. no. RPN 6302 V; GE Healthcare, Chicago, IL, US) at room temperature for 30 min in the dark.

Trypsin (cat. no. V511A, Promega, Madison, WI, US) was added at a final enzyme: protein ratio of 1:100 (w/w) for 16 h at 37 °C. After digestion, 4 μL of 5% trifluoroacetic acid (TFA, cat. no. 302031; Sigma-Aldrich) was added to the samples and incubated at 37 °C for 90 min for Rapigest hydrolysis. The tryptic peptides were centrifuged at 16 000 × *g* for 10 min at 4 °C, and the supernatant was transferred to a new tube and concentrated at 4 °C in a CentriVap concentrator (Labconco, Kansas City, MO, US). The peptides were resuspended and desalted using a C18 ZipTip (cat. No. ZTC18S960, Millipore, Billerica, MA, US) according to the manufacturer’s instructions. The final volume for each sample was obtained by adding an aqueous solution containing 0.1% formic acid (FA) in mass spectrometry (MS) grade water (cat. no. 27001, Sigma-Aldrich) to obtain a final concentration of 200 ng.μL^–1^ of peptides.

### LC-MS/MS

The samples were analyzed using a NanoElute system (Bruker Daltonics, Bremen, Germany) coupled with a hybrid timsTOF Pro mass spectrometer (Bruker Daltonics) equipped with a captive nanoelectrospray source operated at 1500 V.

Chromatographic separation was performed using an Aurora Series UHPLC C18 column (250 mm × 75 μm, 1.6 μm, 120 Å pore size) (IonOpticks, Fitzroy, Australia). Mobile phase A consisted of 0.1% FA in Milli-Q water, and mobile phase B consisted of 0.1% FA in acetonitrile (ACN). The flow rate was set to 300 nL·min^–1^. Peptides were eluted on a stepped gradient of 50 min ramped from 2% to 30% of B (from 2% to 13% over 30 min, 13% to 20% over 15 min, and 20% to 30% in 5 min), plus 10 min of column cleaning (85% B). The data were acquired using the DIA-PASEF method^48^ in the positive ion mode. The parameters were optimized using a data-dependent acquisition parallel accumulation-serial fragmentation (DDA-PASEF) run, acquired from a pooled sample of the analyzed specimens (Supplemental Methods). The isolation width was set at 26 *m*/*z* units, monitoring precursor ions ranging from 315 to 1616 *m*/*z* and ion mobility from 0.66 to 1.50 V·s/cm^2^ (1/K0) across 52 windows, with a cycle time of 0.90 s. The collision energy was linearly ramped from 20 to 59 eV. The tims elution voltage was calibrated linearly to achieve precise 1/K0 ratios using three ions from the ESI-L Tuning Mix (Agilent, Santa Clara, CA, USA) with ions of *m*/*z* 622, 922, and 1222. The calibration occurred before each run, using the’Automatic Calibration’ feature in the control software (timsControl, Bruker).

#### Spectral Library Generation and Data Processing

A spectral library was generated using a previously described method.^49^ We used raw (.d) files from 36 individual in-house saliva runs, including a pooled sample comprising saliva samples (*n* = 38) from our study, all acquired in DDA-PASEF mode. The raw data from five projects in the PRIDE database (PXD036969, PXD033493, PXD023175, PXD029547, and PXD026401) were also incorporated into the library creation.

We employed the label-free quantification match-between-runs (LFQ-MBR) workflow within FragPipe (version 20.0), utilizing MSFragger (version 3.8), Philosopher (version 5.0), and EasyPQP (version 0.1.37) components. The input data consisted of the previously mentioned raw (.d) files, which were searched against a FASTA database (Homo sapiens, taxonomy_id:9606, UniProtKB/Swiss-Prot) downloaded in August 2023. This database contained 20,434 protein sequences and 20,434 (50%) reverse sequences as decoys.

MSFragger’s standard search parameters were applied, with modifications: precursor and fragment mass tolerances were set to 20 ppm and 0.05 Da, respectively; trypsin as enzyme; 7–50 peptide lengths and *m*/*z* for peptide ions were ranged from 500 to 5000. Enzyme specificity was “strict trypsin,″ allowing fully enzymatic peptides and up to two missed trypsin cleavages. The isotope error was set to 0/1/2. Variable modifications included methionine oxidation and protein N-terminal acetylation, with cysteine carbamidomethylation as the fixed modification. Default Philosopher toolkit options, including ProteinProphet, were employed, and results were filtered at a 1% false discovery rate (FDR) at the protein level.

The generated library, containing 2022 proteins and 43151 precursors, was used in DIA-NN (version 1.8.1) to analyze DIA-PASEF runs. The DIA-NN parameters included missed cleavages up to three, allowing up to two variable modifications, and a precursor charge range from 2 to 4; *m*/*z* for precursor ions ranged from 300 to 1800. Default settings were maintained for all other DIA-NN parameters. The DIA-NN was set to optimize the mass accuracy automatically using the first run in the experiment, with output filtered at 1% FDR at precursor and protein levels.^49^ Data were searched against a fasta database (Homo sapiens, taxonomy_id:9606, UniProtKB/Swiss-Prot) downloaded in August 2023. To eliminate potential contaminants, the FASTA file from Frankenfield et al.^50^ was used as a reference. The spectral library and raw data are available via the ProteomeXchange Consortium with the identifier PXD054133.

#### Statistical Analysis, Feature Selection, and Model Classification

Proteomics data analysis was performed using Perseus^51^ and R version 4.3.0 (R Core Team, 2021). The .tsv matrix with the proteins identified by DIA-NN was imported into Perseus (version 2.0.10). Valid values for 70% of the samples from at least one group were retained in the final data matrix. Data were normalized to the Total Ion Chromatogram (TIC) using an in-house R script. Normalized data were imported into Perseus and transformed into log 2(x). Missing values were imputed based on a normal distribution (width = 0.3, downshift = 1.8). A two-sided Student’s *t* test was performed to identify differentially expressed proteins (DEPs) (log 2 Fold Change (FC) ≥ 1.2, FDR < 0.01) in the groups analyzed. To illustrate significant differences in protein expression levels, we generated a volcano plot. Hierarchical clustering analysis (heatmap) using the Z-score of normalized protein abundance was also built in R using gplots, RcolorBrewer, and preprocessCore packages.^52−54^

Feature selection was performed using the Boruta algorithm implemented in R.^55^ A data matrix comprising levels of raw protein abundance was used, ensuring a minimum of 70% valid values. All missing values were replaced with zeros. Random seed values of 648 and a maximum of 650 runs were used to ensure the robustness of the selection process.

To select the best set of proteins for COVID-19 classification, a multivariate exploratory receiver operating characteristics (ROC) analysis was performed with Monte Carlo cross-validation (MCCV) within the “Biomarker Analysis” module of the web platform MetaboAnalyst 6.0 (https://www.metaboanalyst.ca/MetaboAnalyst/).^56^ Overlapping DEPs between Student’s *t* test and Boruta feature selection were used as inputs. Before the analysis, the raw data were log-transformed, and the Support Vector Machine (SVM), Random Forest (RF), and Partial Least Squares-Discriminant Analysis (PLS-DA) algorithms were tested as classification and feature ranking methods. The best model was selected based on a large area under curve (AUC) value. Model performance was evaluated using sensitivity, specificity, balanced accuracy, positive predictive value (PPV), and negative predictive value (NPV) metrics, based on the confusion matrix.

Categorical clinical and demographic variables from the volunteers were analyzed using Fisher’s or chi-square tests. The normality of continuous variables data was assessed using the Shapiro-Wilk test. For normally distributed data, the Student’s *t* test was employed, whereas the Mann–Whitney U test was used for non-normally distributed data.

#### Functional Enrichment and Molecular Complex Detection

Gene Ontology (GO) Analysis for DEPs was performed using the DAVID Platform.^57^ All human genes were used as an enrichment background. Redundant terms were filtered using Revigo.^58^ Enriched pathway analysis was performed using the Reactome Pathway database.^59^ The dot plot for the top ten most enriched pathways (based on FDR values) was plotted using the SR plot platform.^60^

To explore the connection between DEPs, a protein–protein interaction (PPI) network analysis was performed using STRING (v 12.0) (https://string-db.org/). The cutoff score for PPI network analysis was set to 0.7 (high confidence). The PPI network was then exported to Cytoscape (version 3.10.1), and the Molecular Complex Detection (MCODE) plug-in was used to select the main molecular complexes (modules) in the PPI network.^61^ The cutoff criteria were set as follows: “Degree cutoff = 2″, “node score cutoff = 0.2″, “k-core = 2″, and “max. depth = 100”. Functional enrichment of the biological processes, Kyoto Encyclopedia of Genes and Genomes (KEGG), and Reactome pathways were performed for each module. The top three terms were selected based on FDR values.

The COVIDpro database (https://www.guomics.com/covidPro/, accessed on September 24, 2024)^62^ was used to compare our data with 41 COVID-19 proteome data sets. The data set’s identities were provided along with their references.

## Results

### Clinical Characteristics

The study population included 38 participants: 22 with a positive and 16 with a negative RT-qPCR test result. In the COVID-19-positive group, sequencing analysis revealed three SARS-CoV-2 Omicron sublineages: BA.1 (*n* = 9), BA.2 (*n* = 6), and BA.4/BA.5 (*n* = 7). Since the BA.4 and BA.5 subvariants bear identical mutations in the spike (S) protein, they remained undistinguished.

Table 1 shows the clinical and pathological characteristics of the study population. The age and sex distributions for the COVID-19 negative and positive groups were similar (mean age of 45.50 ± 11.38 and 52.09 ± 16.67 years, and Male: Female ratios of 0.60 and 0.57, respectively). At the time of saliva collection, 90.9% of the volunteers who tested positive for COVID-19 and 100% of those who tested negative presented with COVID-19-like symptoms.

**Table 1:** Basic Demographics and Clinical Characteristics of the Study Population^a^

Variable	COVID-19 positive	COVID-19 negative	b*p-value*
Volunteers, n (%)	22 (57.8)	16 (42.1)	0.591
Age (years)	52.09 ± 16.67	45.50 ± 11.38	0.156
Male/Female, n (%)	8/14	6/10	1
Vaccine,^c^ n (%)	7 (31.8)	13 (81.2)	0.006
Comorbidities, n (%)			
Diabetes	3 (13.6)	0	0.248
Kidney disease	2(9)	0	0.499
Immunosuppression	8(36.3)	0	0.011
Heart disease	2(9)	0	0.499
Onset of symptoms to sample collection (days)	4.14 ± 2.34	3.94 ± 1.65	0.916
Symptoms, n (%)			
Cough	16 (72.7)	6 (37.5)	0.047
Coryza	9 (40.9)	9 (56.2)	0.511
Fever	8 (36.6)	5 (31.5)	1
Sore throat	7 (31.8)	7 (43.7)	0.510
Headache	6 (27.2)	4 (25)	1
Myalgia	3 (3.6)	0	0.248
Weakness	2 (9)	0	0.499
Nasal congestion	2 (9)	9 (56.2)	0.002
Ageusia	1 (4.5)	4 (25)	0.140
Sneezing	1 (4.5)	7 (43.7)	0.005
Fatigue	1 (4.5)	2 (12.5)	0.561
Chills	0	4 (25)	0.024

a Data are presented as mean ± standard deviation or number (percentage).

b *p*-values were calculated using Student’s *t* test (or U test Mann-Whitney) for continuous variables and chi-squared test (or Fisher’s exact test) for categorical variables.

c At least one dose of the vaccine was administered; however, the number of doses was not specified.

The average number of days between symptom onset and the date of sample collection was 3.94 ± 1.65 for symptomatic negative volunteers and 4.14 ± 2.34 for symptomatic positive volunteers. The most frequent symptoms among the COVID-19-positive patients were cough (16/22, 72.7%) followed by coryza (9/22, 40.9%), fever (8/22, 36.3%), sore throat (7/22, 31.8%), and headache (6/22, 27.2%). For the COVID-19-negative volunteers, the most common symptoms were coryza (9/16, 56.2%), nasal congestion (9/16, 56.2%), sore throat (7/16, 43.7%), and sneezing (7/16, 43.7%). Except for cough (*p* < 0.047), nasal congestion (*p* < 0.002), and chills (*p* < 0.024), most symptoms did not differ significantly between the two groups. All volunteers who tested positive for COVID-19 were classified as having mild disease, as they presented only minor respiratory symptoms.

Only volunteers in the positive group had comorbidities, with immunosuppression being the most common (8/22, 36.3%), followed by diabetes (3/22, 13.6%), heart disease (2/22, 9.09%), and kidney disease (2/22, 9.09%). At the time of saliva collection, 57% (20/38) of individuals had taken at least one dose of a vaccine against SARS-CoV-2. This included 81.3% (13/16) of the negative group and only 31.8% (7/22) of the positive group.

### Proteomics Analysis

**Figure 2:**
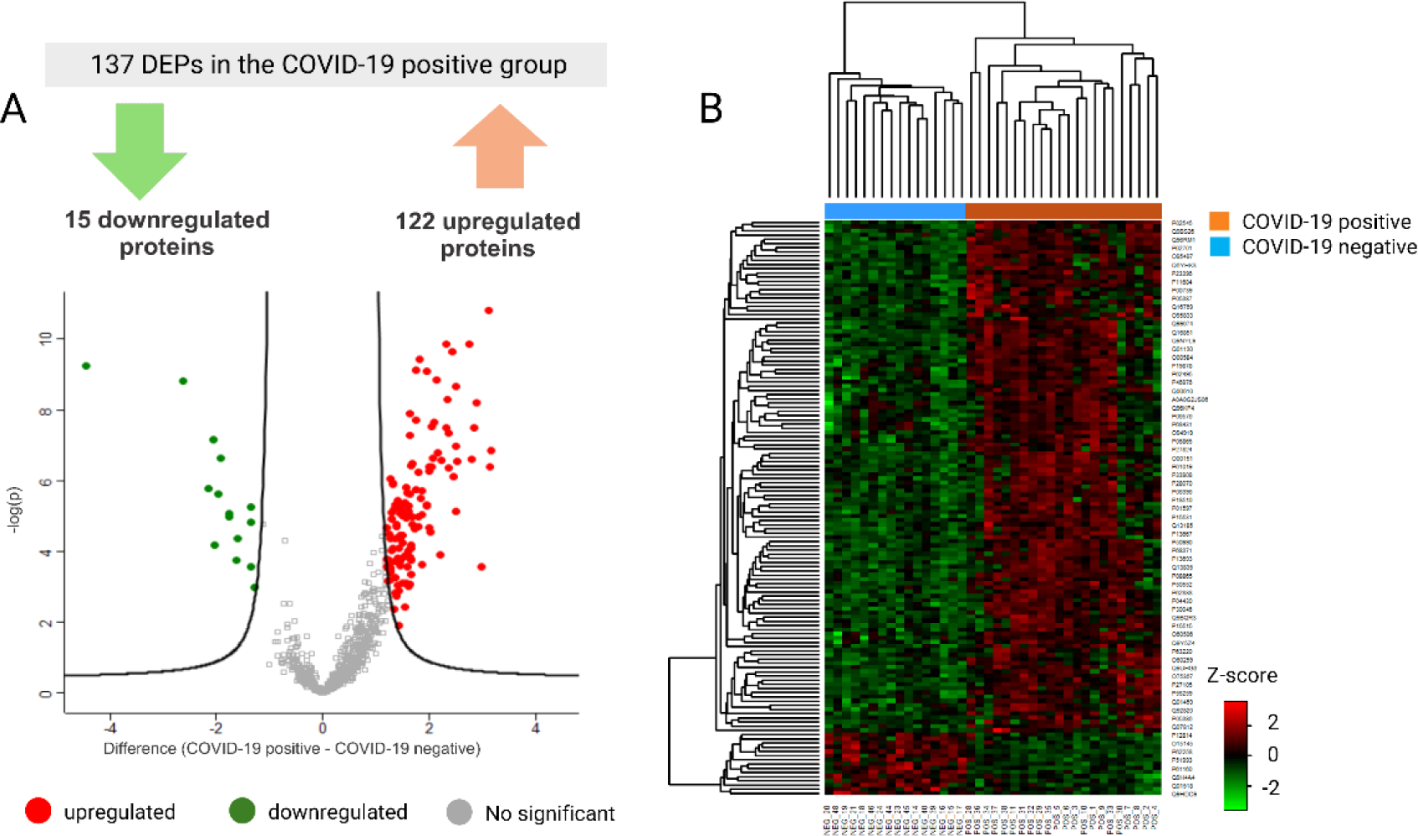
Differentially expressed proteins (DEPs) in the saliva of COVID-19 positive and negative groups. (A) Volcano plot of DEPs showing 122 upregulated proteins (red dots) and 15 downregulated proteins (green dots) in the COVID-19 positive group, as determined by the Student’s *t* test (FDR 1%) with a log 2FC ≥ 1.2. (B) The heatmap shows clusters of samples based on protein expression patterns, with green indicating low and red indicating high differential protein abundance. Z-scores of normalized protein abundances were used, and clustering was performed using Euclidean distance and average linkage. The rows represent the DEPs, while the columns correspond to the individual samples.

Analysis of saliva samples using the DIA-PASEF method retrieved 1194 proteins with 1% FDR. A total of 820 proteins were identified in 70% of the saliva samples from at least one group; of them, 137 proteins were differentially expressed between groups (Supplementary Data 1). Figure 2A displays a volcano plot of the salivary proteomic profiles for the COVID-19 positive vs COVID-19 negative [(log 2FC) vs −log 10 (p-value)], with 122 upregulated proteins on the right (red) and 15 downregulated proteins on the left (green). Figure 2B shows the hierarchical clustering heatmap analysis, which reveals an optimal separation between COVID-19 positive and COVID-19 negative groups based on differential protein abundance.

We applied a multiple-sample test (S0 = 1.5, FDR < 0.05) to evaluate whether different Omicron sublineages (BA.1, BA.2, and BA.4/BA.5) could imprint distinct salivary signatures in patients with COVID-19. Using the Student’s *t* test (log_2_(FC) > 1.2, FDR < 0.05), we also assessed whether the protein profiles of positive volunteers who had received at least one vaccine dose (*n* = 7) differed from those of positive unvaccinated individuals (*n* = 15). These analyses revealed no significant differences between the groups (data not shown). The functional analysis, feature selection, and models were therefore built based only on the SARS-CoV-2 RT-qPCR results.

### Functional Analysis and Signaling Pathways

Gene Ontology analysis of the upregulated proteins in COVID-19-positive samples revealed 83 significant GO terms (*p*-value < 0.05). Figure S1 depicts the top ten biological processes, cellular components, and molecular function terms which are presented from lowest to highest-fold enrichment. GO analysis of proteins exhibiting significant and positive fold change in the COVID-19 positive group revealed enrichment in gene expression regulation processes, including “epigenetic regulation,” “nucleosome assembly,” and “chromatin organization.” These findings highlight key mechanisms associated with gene activity, such as DNA packaging and transcription regulation during SARS-CoV-2 infections (Supplementary Data 2).

Other enriched terms, such as “cytoplasmic translation,” “protein folding,” “protein disulfide isomerase activity,” and “small ribosomal subunits,” suggest potential alterations in protein synthesis efficiency and regulation of the protein synthesis machinery during viral infection. Notably, most of the proteins were localized in the extracellular region or extracellular exosomes, with some cytosolic proteins associated with “focal adhesion,” “nucleosomes,” “melanosomes,” or “macromolecular complexes.” The enrichment of “azurophil granule membrane” proteins also points to critical immune components in saliva (Supplementary Data 2). Pathway enrichment analysis further highlighted “neutrophil degranulation” and the “innate immune system” signaling pathways. Additionally, we identified two enriched pathways—″signaling by ROBO receptors and regulation of SLITs and ROBOs expression”—that were also activated in response to Omicron SARS-Cov-2 infection (Figure S2).

Several signaling pathways and biological processes associated with upregulated proteins related to cell death regulation were also enriched, including “apoptosis,” “regulation of apoptotic processes,” “programmed cell death,” “Chk1/Chk2 (Cds1)-mediated inactivation of the Cyclin B complex”, and “activation of BAD with its translocation to mitochondria,” suggesting an impairment in mechanisms underlying cell death mechanisms (Figures S1, S2 and Supplementary Data 2).

### Molecular Complex Detection

**Figure 3:**
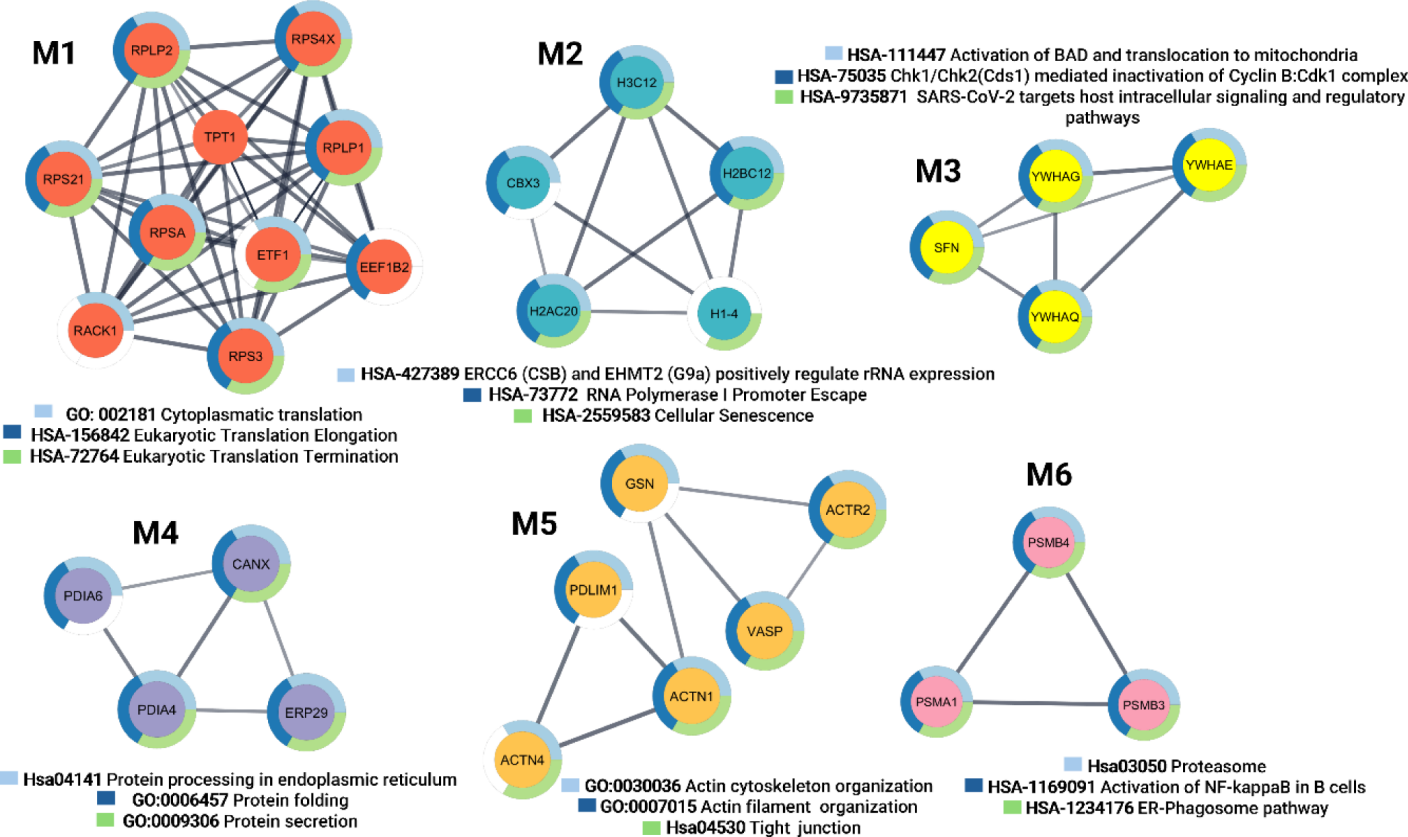
Six densely connected protein complexes obtained from upregulated proteins in the COVID-19 positive cohort using the Molecular Complex Detection (MCODE) algorithm. The top three highly enriched pathways or processes are described for each module. M1, M2, M3, M4, M5, and M6 are modules 1, 2, 3, 4, 5, and 6 respectively.

To gain insights into the protein–protein interaction clusters among the salivary proteins with increased abundance in the COVID-19-positive cohort, PPI and MCODE were also performed. The analyses revealed six highly connected molecular complexes (modules) (Figure 3 and Supplementary Data 3). Note that module 1 comprised ten proteins, including four small ribosomal subunit proteins (RPS21, RPSA, RPS3, and RACK1), two large ribosomal subunit proteins (PRLP2 and RPLP1), and three highly related proteins (ETF1, TPT1, and EEF1B2). The M1 proteins were mainly involved in cytoplasmic translation, eukaryotic translation elongation, and termination of eukaryotic translation.

The second module (M2) was composed of five structural constituents of chromatin: chromobox protein homologue 3 (CBX3), histone H2B type 1-K (H3C12), histone H2A type 2-C (H2AC20), histone H1.4 (H1–4), and histone H3.1 (H3C1). The top three enriched processes were the activation of rRNA expression by ERCC6 (CSB) and EHMT2 (G9a), RNA Polymerase I Promoter, and cellular senescence.

Module 3 (M3) consisted of four proteins from the 14-3-3 protein family: 14-3-3 protein gamma (YWHAG), 14-3-3 protein theta (YWHAQ), 14-3-3 protein epsilon (YWHAE), and 14-3-3 protein sigma (SFN). These proteins are involved in the activation of BAD and its translocation to the mitochondria and Chk1/Chk2(Cds1)-mediated inactivation of the Cyclin B: Cdk1 and have been proposed to be part of the host signaling and regulatory pathways targeted by SARS-CoV-2.

Module 4 (M4) was also composed of protein disulfide-isomerase A6 (PDIA6), protein disulfide-isomerase A4 (PDIA4), endoplasmic reticulum resident protein 29 (ERP29), and calnexin (CANX), which are involved in protein processing in the endoplasmic reticulum, protein folding, and secretion.

Module 5 (M5) included gelsolin (GSN), alpha-actinin-1 (ACTN1), alpha-actinin-4 (ACTN4), PDZ and LIM domain protein 1 (PDLIM1), vasodilator-stimulated phosphoprotein (VASP), and actin-related protein 2 (ACTR2). This module was enriched for actin cytoskeleton organization, actin filament organization, and tight junctions.

Module 6 (M6) comprised three proteasomal proteins: proteasome subunit alpha type-1 (PSMA1), proteasome subunit beta type-3 (PSMB3), and proteasome subunit beta type-4 (PSMB4). These proteins are involved in the proteasome pathway, activation of NF-kappaB in B cells, and ER phagosome pathways.

### Feature Selection and Classification Model

**Figure 4:**
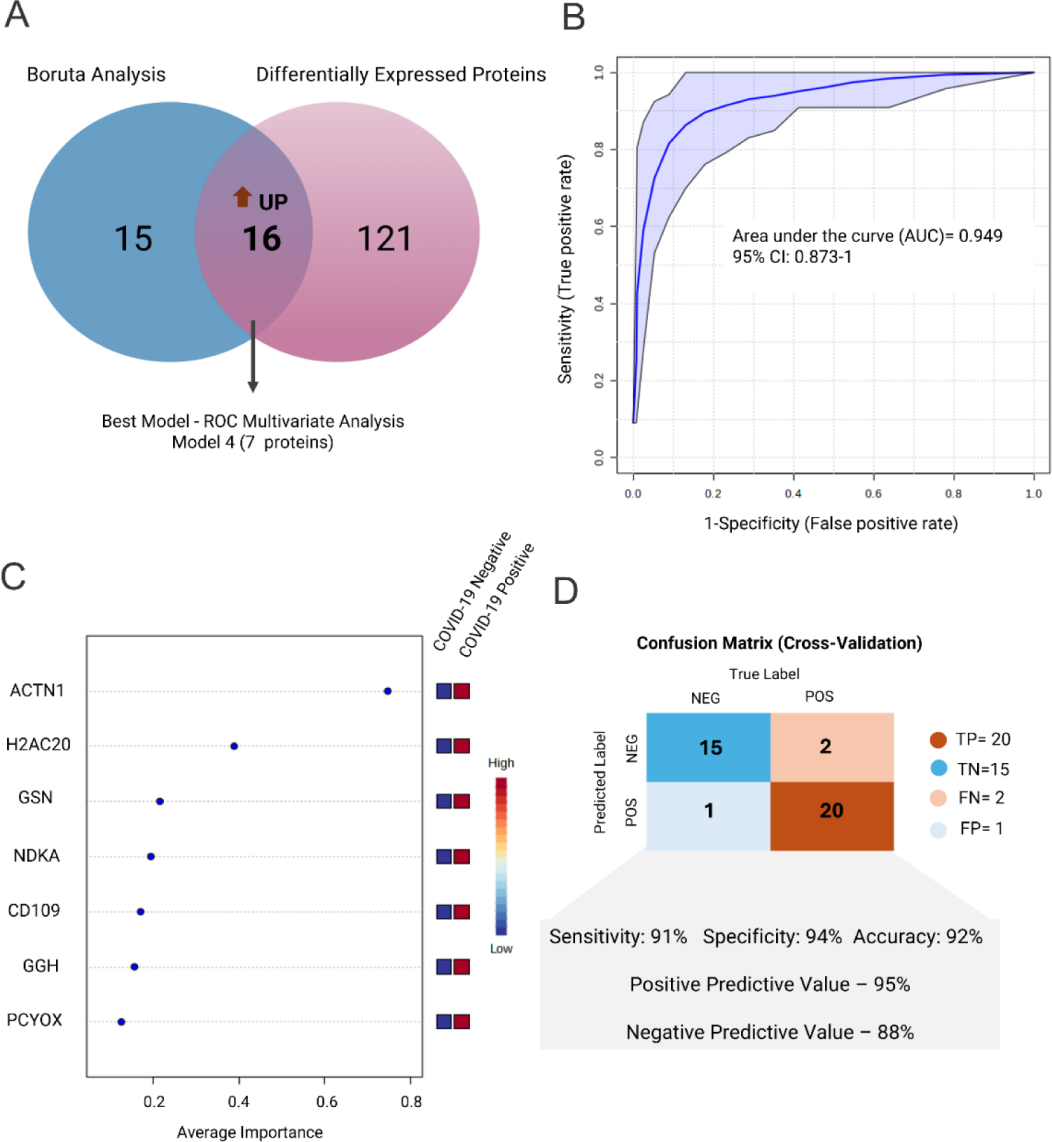
Predictor selection and classification model. A) Venn Diagram indicating the 16 overlapping proteins between Boruta feature selection and differential abundance analysis. B) ROC curve of the best model based on SVM multivariate ROC curve analysis. Model 4 achieved the highest AUC (0.959, CI. 0.873-1). C) Seven selected proteins from Model 4 ranked by average importance. D) Confusion matrix and performance of Model 4 highlighting sensitivity (91%), specificity (94%), balanced accuracy (92%), PPV (95%), and NPV (88%) metrics.

Feature selection using Boruta identified 31 predictors. Only 16 out of 31 predictors were differentially expressed and input to build a classification model to differentiate COVID-19 positive from the negative group (Figure 4A). Table S1 lists all selected proteins. SMV provided the best classification and feature-ranking results among the algorithms tested. The best model, based on multivariate ROC curve analysis, comprised seven proteins: alpha-actinin-1 (ACTN1), histone H2A type 2-C (H2AC2), gelsolin (GSN), nucleoside diphosphate kinase A (NDKA), CD109 antigen (CD109), gamma-glutamyl hydrolase (GGH), and prenylcystine oxidase 1 (PCYOX). Figure 4B shows the model achieved an AUC of 0.949 (confidence interval (CI) of 0.873–1), whereas Figure S3 shows the ROC curves and AUC values from the remaining six biomarker models, considering different numbers of variants. All selected proteins showed increased abundance in the COVID-19-positive group, as Table 2 highlights. Figure 6C displays the predictors of the selected model ranked by order of importance.

**Table 2:** Proteins from The Panel with The Highest AUC in ROC Analysis^a^

Predictors		SVM model	Student’s *t* test		Boruta	Differential expression
Protein Accession	Protein name (Gene)	Importance	log 2(FC)	q-value	normHits	Regulation in COVID-19 pos
P12814	Alpha-actinin-1 (ACTN1)	0.747	1.65	5.11 × 10^–4^	0.98	UP
Q16777	Histone H2A type 2-C (H2AC20)	0.389	1.62	6.80× 10^–4^	0.76	UP
P06396	Gelsolin (GSN)	0.215	1.29	0	1.00	UP
P15531	Nucleoside diphosphate kinase A (NDKA)	0.171	1.30	6.25× 10^–5^	0.54	UP
Q6YHK3	CD109 antigen (CD109)	0.126	1.51	5.88 × 10^–5^	0.51	UP
Q92820	Gamma-glutamyl hydrolase (GGH)	0.194	2.32	0	0.55	UP
Q9UHG3	Prenylcysteine oxidase 1 (PCYOX)	0.156	1.35	6.67 × 10^–5^	0.67	UP

a SVM: support vector machine; FC: fold change; UP: upregulated.

The model correctly classified 20 out of 22 cases as positive for COVID-19 and 15 out of 16 volunteers as COVID-19 negative. These values translates to a sensitivity of 91% and a specificity of 94%. The model also reached a balanced accuracy of 92%, and PPV and NPV of 95% and 88%, respectively (Figure 4 D).

## Discussion

Host response to infectious diseases is influenced by many factors, including patient condition and pathogen characteristics, which affect the course and outcome of the infection.^63^ For COVID-19, as the virus mutates and population immunity becomes widespread, the clinical manifestations, outcomes, and management of the disease have changed significantly compared with that during the initial waves.^64^

To understand disease and potential molecules for therapeutic application, host response remains a crucial factor. Previous efforts have been made to understand the host response to Omicron infections.^19,20,20,65^ However, to the best of our knowledge, this is the first study that demonstrates the salivary protein signature of patients with COVID-19 infected with SARS-CoV-2 Omicron variants. We applied proteomic and functional enrichment combined with feature selection and ROC multivariate analysis to identify dysregulated salivary proteins closely related to mild COVID-19 in patients infected with Omicron subvariants. We then developed a model containing seven proteins that achieved excellent performance in screening COVID-19 in hospital settings.

Earlier salivary studies, with sample collection ranging from April to December 2020, have primarily focused on markers of disease severity, salivary dysregulation in susceptible versus nonsusceptible groups, and the salivary profiles during the convalescent phase of COVID-19.^21,22,25,66^ Key findings included salivary signatures associated with protective antiviral functions,^21,25,66^ and disruptions in sensory taste perception, primarily attributed to cystatin dysregulation.^21,66^ Discriminatory salivary profiles associated with COVID-19 were also linked to inflammatory responses, cell cycle progression, and nucleosome functions.^25^ Impaired innate immunity has also been reported,^66^ and during the convalescent phase, salivary host profiles further highlight ongoing damage characterized by persistent activation of innate immune pathways.^22^

Other salivary proteomic studies, with unreported sample collection dates, revealed alterations in proteins related to the immune system, energy metabolism, protein transport, signal transduction, and apoptosis during SARS-CoV-2 infection.^23^ Pagani et al. found that the salivary host response varies with disease severity, showing significant dysregulation in proteins involved in neutrophil activation, blood coagulation, complement activation, and inflammation.^67^ Recently, Weber et al. reported that salivary extracellular vesicles in COVID-19 patients were linked to an anti-COVID-19 response, highlighting an enrichment in the immune response, oxygen transport, and antioxidant mechanisms.^24^

Herein, we highlight and discuss the key salivary proteins, enriched biological processes, and signaling pathways that were significantly dysregulated in COVID-19 patients mildly infected with the Omicron variants of SARS-CoV-2 from January to July 2022.

### Gene Expression, Protein Metabolism, and Cell Death Mechanisms

Our data revealed that host salivary signatures following Omicron infection were marked by epigenetic gene regulation, protein processing (including translation, internationalization, and folding), attenuated endoplasmic reticulum (ER) stress, and antiapoptotic signaling. These signatures were present in 4 of 6 molecular complexes (M1, M2, M3, and M4). Notably, many SARS-CoV-2 proteins were previously identified as interacting with host proteins involved in these processes.^68^

Our findings align with those of previous reports showing that SARS-CoV-2 restructures the host chromatin architecture. RNA viruses also use this strategy in immune cells to modulate host antiviral defense.^69^ SARS-CoV-2 Omicron infection also affects ribosomal proteins. The ribosome machinery is preferentially modulated by viral pathogens during infections.^70^ This pattern has also been shown for previous SARS-CoV-2 lineages and depends on disease severity.^68^ However, a blood transcriptome profile showed that ribosomal pathways, linked to increased RNA viral translation, were mainly upregulated for Delta and Omicron infections when compared to wild-type SARS-CoV-2 strains,^71^ corroborating our results.

In our analysis, module 1 highlighted 10 ribosomal proteins, 9 of which are also involved in eukaryotic translation elongation and the Sec61 translocon complex. Interestingly, 7 of the 10 proteins in this module were also associated with viral mRNA translation and the Sec61 translocon complex, where several SARS-CoV-2 proteins are inserted into the ER.^72^ This response was also associated with the enrichment of biological processes related to cytoplasmic translation, protein folding, and secretion, as highlighted in modules 1 and 4. Module 4 included four proteins involved in protein processing in the ER and protein folding. Of these, PDIA4, PDI6, and CLX are also involved in ER stress response.

The cellular response to ER stress, known as the unfolded protein response (UPR), is another strategy used by coronaviruses to boost replication and suppress host innate immunity.^73^ ER stress and UPR activation are major triggers of endothelial dysfunction,^74^ related to SARS-CoV-2 and COVID-19 pathogenesis.^75^ The viral protein ORF8 interacts with ER chaperones, intensifying ER stress and UPR, but ORF8 inhibition can mitigate these effects.^76^ Notably, ORF8 is absent, truncated, or expressed at reduced levels in some Omicron subvariants,^77^ which are associated with lower viral loads and milder symptoms in infected patients.^78^

Sustained activation of the UPR or failure to restore ER homeostasis results in cell death via the activation of apoptotic pathways.^79^ In this study, we identified that the abundance of disulfide isomerase A6 increases in the saliva of patients positive for COVID-19. This protein negatively regulates the UPR,^80^ which may suggest better control of ER stress and cell death. Our results support this hypothesis. Regulation of apoptosis and programmed cell death pathways was enriched in our findings, with a notably increased expression of the apoptosis regulator BAX and at least six negative regulators of apoptosis, such as four 14-3-3 proteins, ACTN1, and ACTN4, suggesting a shift toward antiapoptotic signals,^81−83^ which may be associated with the low-severity disease phenotype.^84^

### Salivary 14-3-3 Proteins

Upregulated salivary 14-3-3 proteins in Omicron infection also highlight SARS-CoV-2 targeting of host signaling pathways. To our knowledge, and as module M3 indicates, we are the first to report an increase of four isoforms of the 14-3-3 proteins (sigma, gamma, epsilon, and theta) in the saliva of patients with COVID-19.

The 14-3-3 domain proteins are phospho-binding proteins that regulate key cellular functions, including cell cycle control, apoptosis, signal transduction, energy metabolism, and protein transport.^85^ The interaction between human 14-3-3 proteins and the nucleocapsid protein (N protein) of SARS-CoV-2 is a critical host–virus interaction.^86^ Mutations in N protein enhance its binding to 14-3-3 proteins, thereby improving viral replicative fitness.^87^ This is particularly relevant for Delta and Omicron variants, which exhibit high mutation rates in a mutational hotspot of the N protein with high affinity for 14-3-3 protein isoforms.^87,88^ These mutations have been associated with increased viral infectivity and RNA packaging efficiency.^88,89^

The 14-3-3 proteins are also crucial in pathogen recognition and innate immunity intracellular signaling by regulating the Toll-Like Receptors (TLR) and RIG-I-like Receptor (RLR) signaling pathways.^90^ But whereas the 14-3-3/N interaction may regulate the nucleocytoplasmic transport of the N protein, it represents a potential pathway for cellular hijacking by the virus.^86^ This interaction can impair the 14-3-3 protein function and its interactions with effector proteins, affecting cell cycle and apoptosis, inhibiting innate immunity, and promoting viral replication.^90^

### Immune System

Based on a previous plasma metabolome study, Omicron infections appear to otherwise promote innate immunity over humoral immunity.^19^ Convalescent patients infected with Omicron variants showed an increase in low-density neutrophils and exhibited IL-1β-associated and IFN-responsive signatures.^91^ Immune abnormalities caused by Omicron variants can also persist and underlie long-term symptoms.^19,91^ For instance, exaggerated neutrophil degranulation can contribute to the severity of COVID-19 and potentially affect the development of postacute sequelae of SARS-CoV-2 infection.^92^ But, compared to the original SARS-CoV-2 strain, Omicron infections significantly reduced neutrophil counts and were associated with weaker neutrophil functions.^19,93^ Noteworthy is that salivary proteins in the COVID-19-positive group were shown to be significantly enriched in pathways related to the innate immune system and neutrophil degranulation. This host response was expected since it plays a crucial role in the immune response to viral infection.^94^ Dysregulated innate immunity and neutrophil activation have also been identified as key features of the COVID-19 salivary signature.^23,66,67^ It has also been suggested that COVID-19 progression toward more severe disease may partially result from impaired oral innate immunity.^66^

Pathways related to signaling by ROBO receptors and the regulation of SLIT and ROBO expression were also enriched in the positive cohort, as represented by 12 upregulated proteins of our study, which is the first report of this finding in saliva. Interestingly, SLIT/ROBO signaling proteins inhibited leukocyte chemotaxis toward chemoattractants *in vitro* and *in vivo* models.^95^ The Robo-4-dependent signaling pathway also strengthened the vascular barrier and reduced the harmful effects of the host response to pathogen-induced cytokine storms in an animal model of polymicrobial sepsis and H5N1 influenza.^96^ Robo4 expression has also been linked to reduced vascular permeability and decreased mortality during severe infectious diseases, including COVID-19.^97^ In line with these findings, Merchant et al. reported that genes involved in inflammatory and immune responses, mainly chemokine signaling and viral protein interaction with cytokines, were suppressed in Omicron infection.^71^ Collectively, the data suggests that the upregulation of SLIT/ROBO signaling proteins represent a potential compensatory mechanism that may be activated in response to impaired innate immune during Omicron SARS-Cov-2 infection. This response can also be linked to mild disease phenotype in Omicron infections in our positive cohort, warranting additional research in this area.

### Potential Salivary Protein Signature for COVID-19 Screening and Insights into Disease Severity

We evaluated the salivary proteome performance in discriminating mild COVID-19 patients from the negative cohort in a hospital setting. The optimal proteomic-driven panel includes seven predictors: ACTN1, H2AC2, GSN, NDKA, CD109, GGH, and PCYOX. This panel shows great potential for translational applications, achieving 91% sensitivity and 94% specificity. While most previous studies on saliva for COVID-19 screening and diagnosis primarily focused on viral protein detection,^98−100^ our study focused on host proteins to discriminate between COVID-19 positive and negative groups. Notably, this panel was able to differentiate patients with similar symptoms, which is particularly challenging but crucial in hospital settings due to the presence of vulnerable individuals.

We then sought to explore the connection between the highlighted proteins and potential SARS-CoV-2 pathophysiological mechanisms. We also investigated how the abundance levels of these proteins correlated with disease severity in previous salivary reports and other biological fluids (serum, plasma, and urine), using the COVIDPro database.

ACTN1, a cytoskeletal actin-binding protein, interacts with multiple SARS-COV-2 proteins,^101^ and increased ACTN1 expression has been linked to increased cell adhesion, which plays a significant role in the development and progression of Omicron infection.^65^ High levels of ACTN1 in urine and plasma have been associated with nonsevere COVID-19 (Shu_1, Li J._1, Bi_3).^102−104^ Interestingly, increased levels have also been observed in salivary samples from convalescent COVID-19 patients. ^22^

H2AC20 is a core component of nucleosomes and plays a crucial role in transcriptional regulation, DNA repair, replication, and chromosomal stability.^105^ Salivary histone H2A interferes with the access of SARS-CoV-2 to ACE2-expressing host cell*s in vitro* by masking ACE2 and inhibiting viral entry.^106^ Interestingly, when compared to healthy and nonsevere COVID-19, serum abundance levels of H2AC20 proteins were upregulated in severe disease (Bi_1, Shen_1).^104,107^

GSN is a regulatory protein involved in actin filament polymerization.^108^ Beyond its cytoplasmic functions, plasmatic gelsolin (pGSN) plays a crucial role in removing cellular debris released from damaged tissues in multiple organs, including those caused by cytokine storms in COVID-19.^109,110^ pGSN also acts as a negative regulator of pro-inflammatory cytokines, highlighting its role in immune regulation.^111^ Reduced serum and plasma GSN levels in COVID-19 patients were associated with disease severity (Demichev_1, 2 and 3, Messner_1, 2 and 3, Shu_1, and 2),^102,110,112^ and poor clinical outcomes.^41,42^ Salivary GSN levels were downregulated in symptomatic, but not asymptomatic, patients infected with wild-type SARS-CoV-2 strains.^66^ In contrast, we detected increased salivary GSN levels during Omicron infection. Its abundance in patients with mild illness aligns with previously published serum phenotypes, suggesting a protective effect in the positive cohort, which is indicative of a favorable prognosis.

NDKA maintains the nucleotide pool, including NTP homeostasis,^113^ but the association between NDKA and SAR-CoV-2 infection remains unclear. An *in vitro* study has shown NDKA antiviral activity against the foot-and-mouth disease virus (FMDV). NDKA suppresses viral replication in FMDV-infected cells by enhancing host antiviral response through p53-mediated functions.^114^ Curiously, the p53 apoptosis effector was found in low abundance in the saliva of patients with COVID-19,^23^ indicating the need for more studies in this way.

CD109 is a glycosylphosphatidylinositol-anchored protein and a member of the α2 macroglobulin (α2M)/C3, C4, C5 family.^115^ CD109 acts as a substrate for glucose-regulated protein 78 (GRP78), a cell surface receptor that mediates SARS-CoV-2 recognition and entry into human cells.^116^ Host cell recognition by cs-GRP78 is enhanced in the SARS-CoV-2 Omicron variant.^117^ Interestingly, csGRP78 also forms a complex with CD109 and suppresses TGF-β signaling.^118^ This complexation is particularly relevant since TGF-β signaling is involved in SARS-CoV-2 infection,^119^ COVID-19-associated acute respiratory distress syndrome (ARDS,) and pulmonary fibrosis.^120^ These data suggest that CD109 may inhibit viral entry by acting as a competitive ligand for csGRP78 and that its interaction with GRP78 promotes an anti-inflammatory response. In line with this finding, a lower abundance of plasmatic CD109 has been associated with severe and critical COVID-19 (Shen_1 and Shen_2 data sets).^107^

GGH is a ubiquitously expressed lysosomal enzyme involved in intracellular folate metabolism for cell proliferation, DNA synthesis, methylation, and repair.^121,122^ This enzyme is also involved in neutrophil immune response,^123^ and the human GGH and SARS-CoV-2 proteins interaction was found to down-regulate IL-17 and IFN-α,^124^ also contributing to an anti-inflammatory response. Interestingly, blood GGH levels are upregulated in nonsevere COVID-19 compared to healthy controls and showed a significant tendency to increase as the disease progresses (Bi_1, Lee_1, and Shen_1 data sets).^104,107,125^

PCYOX1 plays a key role in protein prenylation and in the release of hydrogen peroxide.^126^ Urine and serum PCYOX1 levels are negatively regulated in patients with severe and critical COVID-19 (Shen_1 and Shu_1 data sets).^102,107^ Lower PCYOX1 levels in these patients may contribute to COVID-19-associated liver dysfunction.^40^ Its high abundance in the COVID-19-positive cohort suggests potential protective effects in low-severity cases.

Notably, most proteins in the panel were associated with anti-COVID, and antiviral activity reflecting a typical response imprinted in saliva following SARS-CoV-2 infection^24,25^ But only ACTN1 and GSN have previously been reported as dysregulated in the saliva of patients with COVID-19 or convalescent individuals, and NDKA has not been associated with COVID-19 until now. Still, although we did not follow up on disease progression or patient outcome, some of the COVID-19 blood and urine proteome data sets aligned with our results and offered additional insights into the severity of the disease and its prognosis, pointing out saliva as a potential substitute for more invasive biofluids in screening and monitoring the disease.

While our study provides valuable insights into the salivary proteomic signature of patients with COVID-19 upon Omicron variants infection, it is important to address some of its clinical limitations. Hospital recruitment of volunteers may introduce confounding factors, such as comorbidities and medications. Our study also primarily focused on the differences between two symptomatic groups, and additional variations in salivary profiles could emerge when comparing the data with asymptomatic groups. Although our analysis highlighted some promising salivary prognostic markers, clinical outcomes were not evaluated, and the long-term salivary response to Omicron infection was also unassessed, which restricted our conclusions regarding disease progression. Data on vaccination doses were also unavailable, limiting our knowledge of the immunization status of volunteers. Therefore, further validation of the protein panel in a larger cohort of volunteers representing other respiratory infections, disease severity, age groups, and emerging variants is necessary to confirm the robustness of the proposed salivary panel and its clinical applicability.

## Conclusion

We studied the salivary proteome changes in COVID-19 patients after the emergence of the Omicron variants, which uncovered new insights into the host response to SARS-CoV-2 infection with potential implications for the pathophysiology and severity of COVID-19. Salivary signatures were characterized by enhanced ribosomal regulation, consistent with increased protein synthesis and processing, supporting previous findings on Omicron infection. The higher abundance of 14-3-3 proteins in saliva, first reported here, may also be associated with increased infectivity and improved viral replicative fitness of Omicron variants. The enrichment of SLIT/ROBO signaling proteins suggests a potential protective mechanism activated in response to innate immune impairment during Omicron infection, which may contribute to the mild symptoms in the positive cohort.

We also identified seven proteins with good performance in screening COVID-19-positive patients in a hospital setting. These included novel (NDKA) and previously reported biomarkers (ACTN1, H2AC20, GSN, CD109, GGH, and PCYOX1) detected in the plasma and urine of patients with COVID-19. The detection of these key biomolecules across multiple proteome data sets also offered insights into disease severity, further suggesting that saliva could serve as an alternative for disease-related circulating biomarkers, supporting its potential for future translational applications in infectious diseases.

## Data Availability

The mass spectrometry proteomics data have been deposited to the PRIDE Archive (http://www.ebi.ac.uk/pride/archive/) via the PRIDE partner repository with the data set identifier PXD054133.

## Supporting Information Available

**Supplementary data 1:** Differentially expressed proteins (DEPS) in the COVID-19 positive group (XLSX)
**Supplementary data 2**: Gene ontology analysis of upregulated proteins in the COVID-19 positive group (XLSX)
**Supplementary data 3:** Molecular complex detection data (XLSX)
Supplemental method, DDA-PASEF experiment used for the generation of Spectral library; Figure S1: Gene ontology analysis of upregulated salivary proteins in the COVID-19 positive group; Figure S2: Dot plot showing the enriched reactome pathways for the upregulated proteins in the COVID-19 positive group; Figure S3: Biomarker prediction by multivariate ROC curve-based exploratory analysis; Table S1: Metadata of all volunteers recruited in the study; Table S2: List of overlapped differentially expressed proteins between the Student’s *t* test and Boruta feature selection (PDF)

## Abbreviations

AUC: Area under the curve
CI: Confidence interval
DEP: Differentially expressed proteins
FA: Formic acid
FMDV: Foot-and-mouth disease virus
GGH: Gamma-glutamyl hydrolase
GO: Gene Ontology
HUPES: Hospital Universitário Professor Edgard Santos
KEGG: Kyoto Encyclopedia of Genes and Genomes
LAPI: Laboratory of Research in Infectology
MCCV: Monte Carlo cross-validation
MCODE: Molecular Complex Detection
MS: Mass spectrometry
NPV: Negative predictive value
PPI: Protein−Protein Interaction
PPV: Positive predictive value
RBD: Receptor-binding domain
RF: Random Forest
RLR: RIG-I-like Receptor
ROC: Receiver operating characteristic
SVM: Support Vector Machine
TIC: Total Ion Chromatogram
TLR: Toll-Like Receptors
UFBA: Federal University of Bahia
UPR: Unfolded protein response
VASP: Vasodilator-stimulated phosphoprotein
