# Supplementary material for: 4D-DIA Proteomics Uncovers New Insights into Host Salivary Response Following SARS-CoV-2 Omicron Infection
Source: J Proteome Res. 2025 Jan 13;24(2):499–514. doi: 10.1021/acs.jproteome.4c00630 (PMC11812090; doi:10.1021/acs.jproteome.4c00630)
Supplement: Supplementary file 4 — pr4c00630_si_004.pdf [file pr4c00630_si_004.pdf]

Supporting Information for

4D-DIA Proteomics Uncovers New Insights into Host Salivary Response Following SARS-CoV-2 Omicron Infection

Iasmim Lopes de Lima[1], Thais Regiani Cataldi[2], Carlos Brites[3], Mônica Teresa Veneziano Labate[2], Sara Nunes Vaz[3], Felice Deminco[3], Gustavo Santana da Cunha[1], Carlos Alberto Labate[2], and Marcos Nogueira Eberlin[1*]*

[1] PPGEMN, School of Engineering, Mackenzie Presbyterian University & MackGraphe - Mackenzie Institute for Research in Graphene and Nanotechnologies, Mackenzie Presbyterian Institute, São Paulo – SP, 01302-907, Brazil.

2 Department of Genetics, “Luiz de Queiroz” College of Agriculture, University of São Paulo/ESALQ, Piracicaba, SP 13418-900, Brazil

3 LAPI - Laboratory of Research in Infectology, University Hospital Professor Edgard Santos (HUPES), Federal University of Bahia (UFBA), Salvador, BA 40110-060, Brazil

*Corresponding authors: Iasmim Lopes de Lima (iasmim.lima@mackenzista.com.br); Marcos Nogueira Eberlin (marcos.eberlin@mackenzie.br)

Table of Contents

1. **Supplemental Method**

2. **Supplemental Figures**

Figure S1. Gene Ontology analysis of upregulated salivary proteins in the COVID-19 positive group.

Figure S2. Dot plot showing the enriched Reactome pathways for the upregulated proteins in the COVID-19 positive group.

Figure S3. Biomarker prediction by multivariate ROC curve-based exploratory analysis.

3. **Supplemental Tables**

Table S1. Metadata of all volunteers recruited in the study.

Table S2. List of overlapped differentially expressed proteins between the Student's t-test and Boruta feature selection.

Supplemental Method

DDA-PASEF experiment used for the generation of Spectral library

LC-MS analysis of peptides from the pooled sample was performed on a NanoElute (Bruker Daltonics, Bremen, Germany) system coupled online to hybrid timsTOF Pro mass spectrometer (Bruker Daltonics, Bremen, Germany), equipped with a captive nano-electrospray source operated at 1500V.

Approximately 200 ng of peptides were separated on an Aurora column 25 cm × 75 µm ID, 1.9 um reversed-phase column (IonOpticks, Fitzroy, Australia) at a flow rate of 300 nL min^{-1} at 50 °C. Mobile Phase A consisted of 0.1% Formic acid (FA) in MilliQ water, and B comprised 0.1% FA in Acetonitrile (ACN). We used a gradient starting with a linear increase from 2% B to 17% B over 60 min, followed by further linear increases to 25% B in 30 min and 37% B in 10 min. Finally, 95% B was achieved in 10 min and held constant for 10 min. The column was equilibrated using four volumes of solvent A. The mass spectrometer was operated in data-dependent PASEF mode with one survey TIMS-MS and 10 PASEF MS/MS scans per acquisition cycle. We analyzed an ion mobility range from 1/K0 = 1.6 to 0.6 Vs. cm^{-2} using equal ion accumulation and ramp time in the dual TIMS analyzer of 100 ms each. Suitable precursor ions for MS/MS analysis were isolated in a window of 2 Th for *m/z* < 700 and 3 Th for *m/z* > 700 by rapidly switching the quadrupole position in sync with the elution of precursors from the TIMS device. The collision energy was lowered stepwise as a function of increasing ion mobility, starting from 20 eV for 1/K0 = 0.6 Vs cm-2 and 59 eV for 1/K0 = 1.6 Vs cm-2. The ion mobility dimension was calibrated linearly using three ions from the Agilent ESI LC/MS tuning mix (m/z, 1/K0: 622.0289, 0.9848 Vs. cm^{-2}; 922.0097, 1.1895 Vs. cm^{-2}; and 1221.9906, 1.3820 Vs. cm^{-2}).

Supplemental Figures

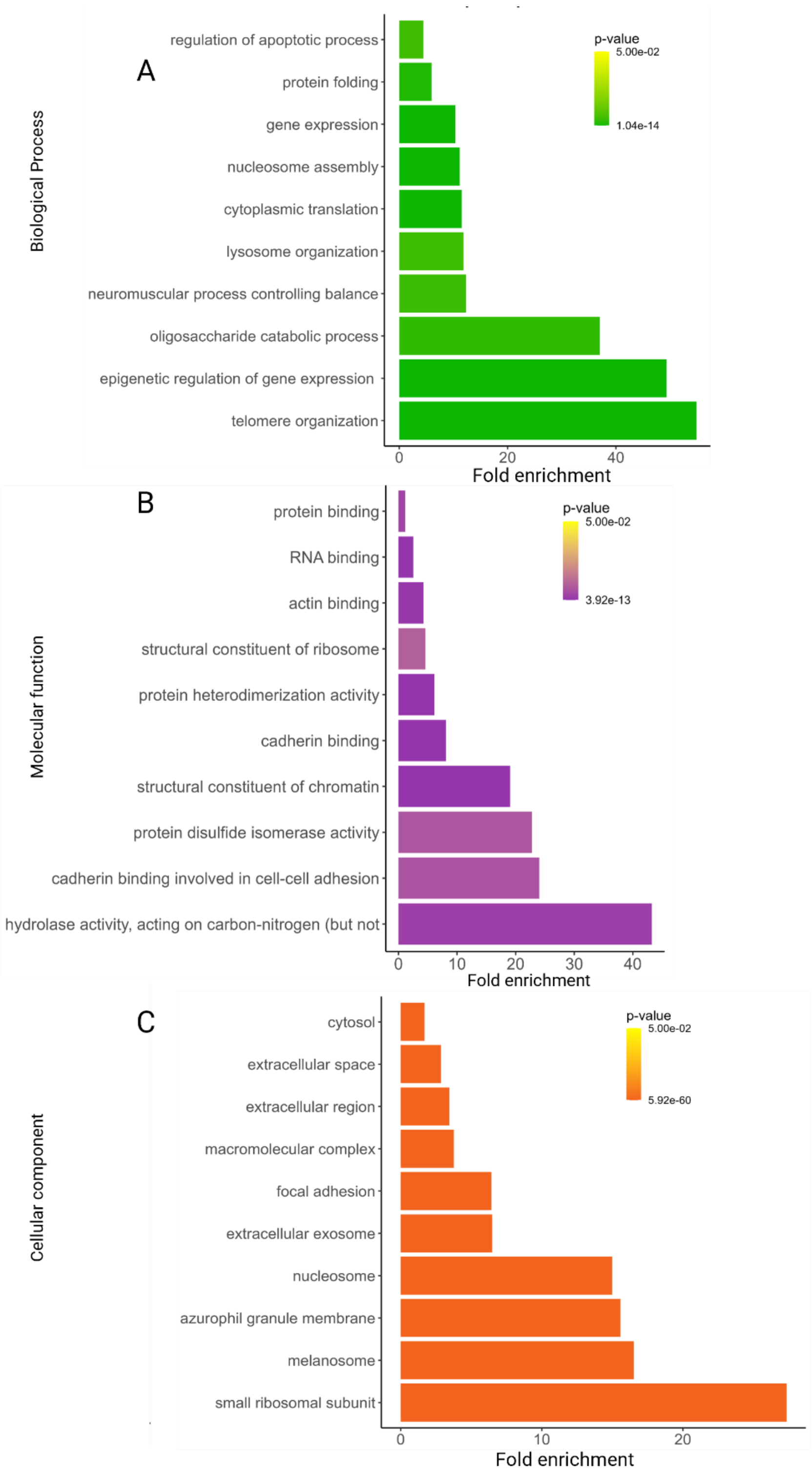

Figure S1. Gene Ontology (GO) analysis of upregulated salivary proteins in the COVID-19 positive group. A) Bar charts showing the top 10 GO terms for Biological process (A), Molecular function (B), and Cellular components (C), ranked by fold enrichment.

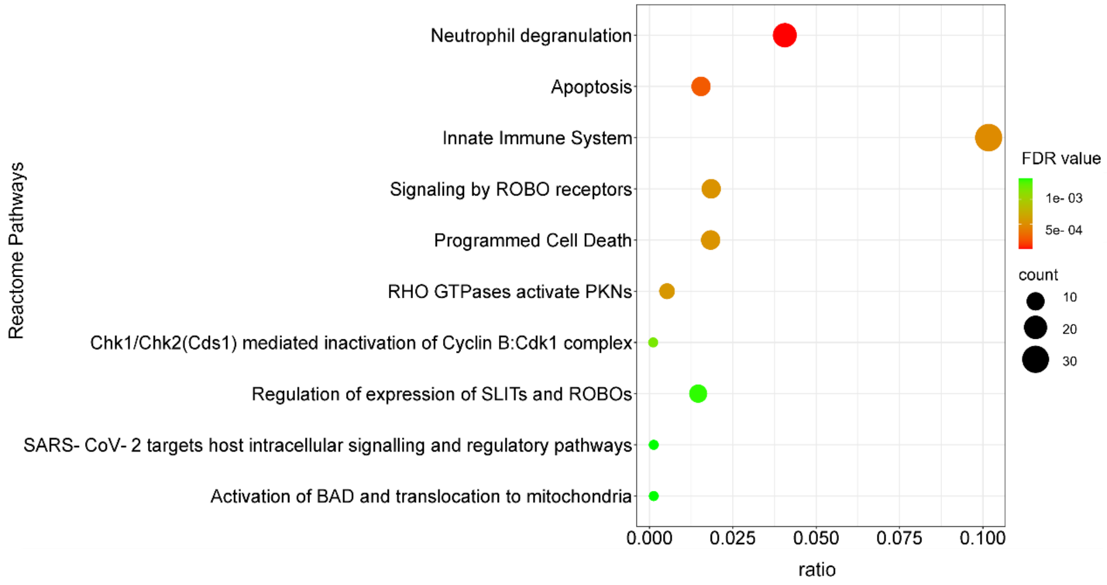

Figure S2. Dot plot showing the enriched Reactome pathways for the upregulated proteins in the COVID-19 positive group. The *x*-axis represents the protein ratio (i.e., the proportion of Reactome pathway molecules represented by this pathway), and the *y*-axis represents the enriched pathways. The dot size is proportional to the protein count. Dot colors represent FDR values.

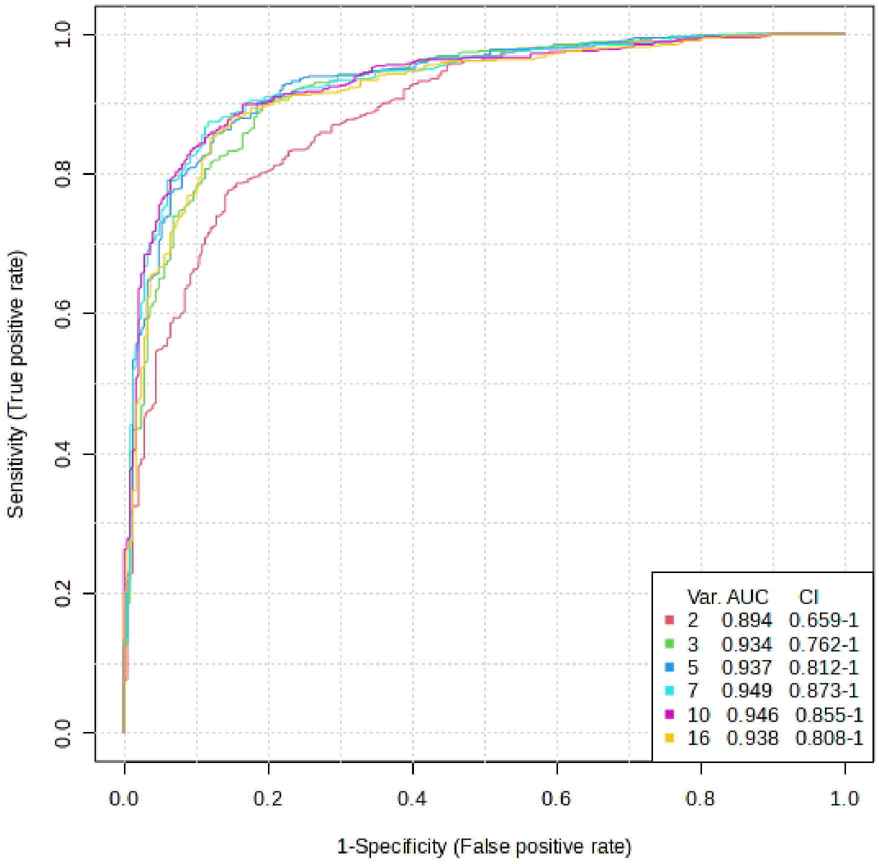

Figure S3. Biomarker prediction by multivariate ROC curve-based exploratory analysis. An Overview of all ROC curves created from 6 different biomarker models considering the different number of variants (2, 3, 5, 7, 10, and 16) with their corresponding AUC value and confidence interval.

Table S1. Metadata of all volunteers recruited in the study.

Patient_ID	RT-qPCR	Age (years)	Sex	Days between SO and SLC*	SARS-CoV-2 variant	ct.value gene E	ct.value gene RDRP
1	Positive	61	F	2	B.1.1.529 (BA.1)	32.7	31.2
2	Positive	36	M	3	B.1.1.529 (BA.1)	30.3	29.8
3	Positive	36	M	3	B.1.1.529 (BA.1)	24.9	23.4
4	Positive	56	F	5	B.1.1.529 (BA.1)	20.9	19.8
5	Positive	44	F	2	B.1.1.529 (BA.1)	34.8	34.1
6	Positive	25	M	2	B.1.1.529 (BA.1)	20.7	19.8
7	Positive	52	M	7	B.1.1.529 (BA.2)	36.6	35.6
8	Positive	60	F	7	B.1.1.529 (BA.2)	25.7	21.4
9	Positive	39	M	3	B.1.1.529 (BA.2)	35.5	35
10	Positive	39	F	6	B.1.1.529 (BA.2)	23.1	22.1
11	Positive	30	M	6	B.1.1.529 (BA.2)	24	23.7
28	Positive	83	F	10	B.1.1.529 (BA.4/BA.5)	22	21.8
29	Positive	76	F	4	B.1.1.529 (BA.4/BA.5)	23.7	23.1
30	Positive	71	F	1	B.1.1.529 (BA.4/BA.5)	29.8	28.4
31	Positive	74	F	3	B.1.1.529 (BA.4/BA.5)	31.1	39.2
32	Positive	59	F	8	B.1.1.529 (BA.4/BA.5)	32.4	31.7
33	Positive	49	F	5	B.1.1.529 (BA.4/BA.5)	32.9	31.2
34	Positive	64	M	4	B.1.1.529 (BA.2)	9	8.9
35	Positive	49	F	3	B.1.1.529 (BA.1)	26.6	25.9
36	Positive	38	F	2	B.1.1.529 (BA.1)	28.9	27.8
37	Positive	33	M	3	B.1.1.529 (BA.1)	31.2	30.1
38	Positive	72	F	2	B.1.1.529 (BA.4/BA.5)	30.9	29.8
14	Negative	33	M	4	NA	-	-
15	Negative	48	M	4	NA	-	-
16	Negative	43	M	6	NA	-	-
17	Negative	34	F	4	NA	-	-
18	Negative	24	F	3	NA	-	-
19	Negative	47	M	3	NA	-	-
20	Negative	34	F	4	NA	-	-
21	Negative	59	M	5	NA	-	-
23	Negative	61	F	2	NA	-	-
24	Negative	40	F	7	NA	-	-
39	Negative	47	F	4	NA	-	-
40	Negative	54	F	2	NA	-	-
44	Negative	51	F	4	NA	-	-
45	Negative	38	F	7	NA	-	-
46	Negative	49	M	2	NA	-	-
48	Negative	66	F	2	NA	-	-

*Days between the onset of symptoms and saliva collection; Ct value; Cycle Threshold value; NA: Non-applicable; RT-qPCR: Reverse transcription-quantitative polymerase chain reaction; SO: symptoms onset; SLC: saliva collection.

Table S2. List of overlapped differentially expressed proteins between the student's t-test and Boruta feature selection.

Accession UniProt	Protein (GENE)	Log(2)FC	q-value	NormHit (Boruta)	Regulation in COVID-19 POS
**O43707**	Alpha-actinin-4 (ACTN4)	1.42	1.08E-03	0.89	UP
**P06396**	Gelsolin (GSN)	1.29	0.00E+00	1.00	UP
**P09211**	Glutathione S-transferase P (GSTP1)	1.21	1.82E-03	0.49	UP
**P12814**	Alpha-actinin-1 (ACTN1)	1.65	5.11E-04	0.98	UP
**P15531**	Nucleoside diphosphate kinase A (NDKA)	1.30	6.25E-05	0.54	UP
**P30040**	Endoplasmic reticulum resident protein 29 (ERP29)	1.62	7.14E-05	1.00	UP
**P30048**	Thioredoxin-dependent peroxide reductase, mitochondrial (PRDX3)	1.51	4.57E-03	0.52	UP
**P37802**	Transgelin-2 (TAGL2)	1.64	1.09E-03	0.58	UP
**Q01518**	Adenylyl cyclase-associated protein 1 (CAP1)	1.25	1.32E-03	1.00	UP
**Q15084**	Protein disulfide-isomerase A6 (PDIA6)	1.68	1.05E-04	0.97	UP
**Q16777**	Histone H2A type 2-C (H2AC20)	1.62	6.80E-04	0.76	UP
**Q6YHK3**	CD109 antigen (CD109)	1.51	5.88E-05	0.51	UP
**Q92820**	Gamma-glutamyl hydrolase (GGH)	2.32	0.00E+00	0.55	UP
**Q9BS26**	Endoplasmic reticulum resident protein 44 (ERP44)	1.40	0.00E+00	0.99	UP
**Q9NYL9**	Tropomodulin-3 (TMOD3)	2.45	0.00E+00	1.00	UP
**Q9UHG3**	Prenylcysteine oxidase 1 (PCYOX)	1.35	6.67E-05	0.67	UP

FC: Fold change.
